# A humanized eNAMPT -neutralizing monoclonal antibody reduces cancer-induced bone pain and osteoclastogenesis in murine breast cancer model

**DOI:** 10.3389/fpain.2026.1955262

**Published:** 2026-10-21

**Authors:** Nikhil C. Mathur, Ethan Villaroel, Isabella Villaroel, Erika Liktor-Busa, Kelly L. Karlage, Manvir Kaur, Carrie Kempf, Beatrice Gudaite, Zuo Ding, Joe G. N. Garcia, Tally M. Largent-Milnes, Todd W. Vanderah

**Affiliations:** 1 Department of Pharmacology, College of Medicine, University of Arizona, Tucson, AZ, United States; 2 The Herbert Wertheim UF Scripps Institute for Biomedical Innovation & Technology, University of Florida, Jupiter, FL, United States

**Keywords:** bone metastasis, bone osteoclastogenesis, breast cancer, cancer-induced bone pain, murine model of pain

## Abstract

**Introduction:**

Cancer-induced bone pain (CIBP) is a common symptom among patients with metastasized cancer, however, health professionals often face challenges in managing patients’ pain. Extracellular nicotinamide phosphoribosyltransferase (eNAMPT) is a DAMP overexpressed in metastatic breast carcinoma that binds to Toll-Like Receptor 4 (TLR4) to potentially trigger a signaling cascade through Nuclear Factor kappa *β* (NFk-*β*) inducing inflammation, osteoclastogenesis, and contributing to CIBP. To assess the impact of eNAMPT on CIBP and to identify alternative therapeutic options, ALT-100, an eNAMPT-neutralizing monoclonal antibody, was tested in murine and cell-based models.

**Methods:**

First, eNAMPT synthesis and secretion by EO771 breast adenocarcinoma cells and its modulation by ALT-100 were assessed using eNAMPT ELISA. Next, recombinant eNAMPT-induced osteoclastogenesis and its inhibition by ALT-100 were evaluated in RAW 264.7 osteoclast precursor cells using TRAP staining. *In vivo*, female C57BL/6J mice underwent intramedullary femoral injection of EO771 cells to establish CIBP or SHAM surgery. One week later, mice received ALT-100 (4 mg/kg, ip) or saline control, followed by longitudinal assessment of pain-related behaviors and femoral radiographs.

**Results:**

The eNAMPT ELISA assay revealed that EO771 cells synthesize and secrete eNAMPT in a time-dependent manner maximal at 48 h, which were reduced by ALT-100 mAb. In RAW 264.7 cells, recombinant eNAMPT significantly increased TRAP activity, while co-administration of ALT-100 significantly reduced this effect. In the murine CIBP model, ALT-100 significantly reduced pain-related behaviors compared to saline control mice in the cancer group. Moreover, radiographic analysis demonstrated that ALT-100 mitigates cancer-induced bone loss.

**Discussion:**

These data show that ALT-100 exhibits antinociceptive effects in a CIBP murine model and inhibits eNAMPT-induced osteoclast differentiation. Our results indicate that blockade of eNAMPT can potentially serve as an alternative clinical strategy for treating CIBP.

## Graphical Abstract

**Figure:**
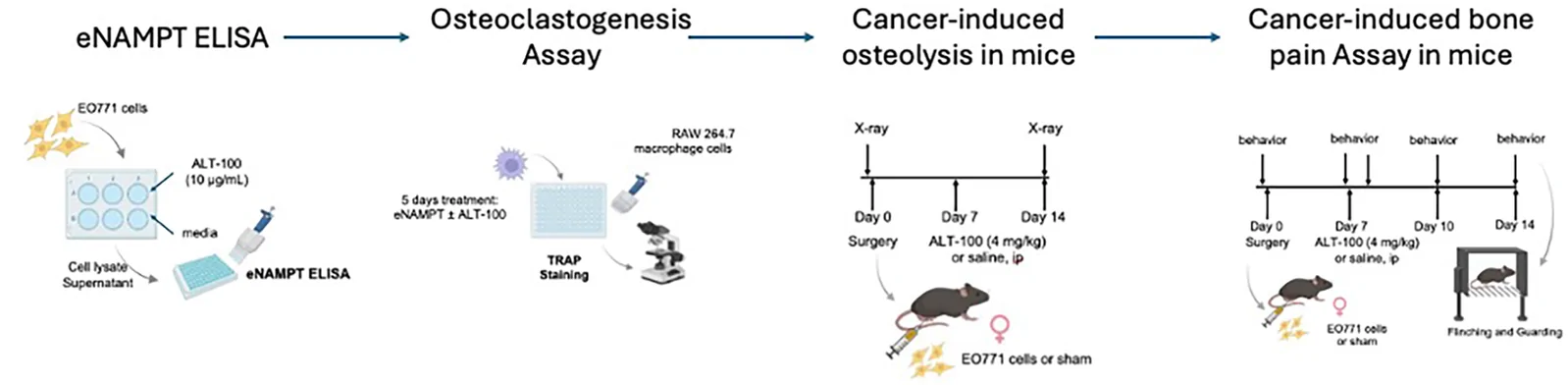
Created with BioRender.com.

## 1 Introduction

Several types of cancer, including breast, prostate, and lung cancer commonly metastasize to bone, which leads to bone destruction and subsequently resulting in a chronic pain condition characterized as cancer-induced bone pain (CIBP) (1). Human data report that 30%–50% of all cancer patients and 75%–90% of late-stage patients experience chronic pain (1–3) CIBP has been characterized as a combination of inflammatory and neuropathic pain with episodes of debilitating “break-through” pain suggesting that current treatment such as high doses of opioids cannot attenuate the pain (3). Opioid-based analgesics are the therapeutic standards to alleviate severe pain caused by cancer metastasis, however they often cause major adverse side effects, like respiratory depression and addiction (4). Studies also show opioids could possibly increase the rate of cancer progression, recurrence, and mortality (5–7) as well as have been shown to potentially increase bone loss (8). There are limited alternative therapeutic strategies to target the progression of cancer-induced bone pain. Thus, there is a need for understanding the inflammatory and neuropathic mechanisms of CIBP in order to develop new, more efficient interventions that lack unwanted side effects.

Recent research identified the role of eNAMPT (extracellular nicotinamide phosphoribosyltransferase) in the development of radiation-induced lung fibrosis (RILF) (9). eNAMPT is a damage-associated molecular pattern protein (DAMP) with high affinity to bind to the pathogen-recognition receptor, Toll-like receptor 4 (TLR4) (10). The eNAMPT/TLR4-triggered inflammatory network dysregulates signaling pathway genes and proteins leading to disruption in fibrogenesis during radiation exposure (9, 11). Previous lung cancer studies have revealed increasing levels of eNAMPT due to radiotherapy (9, 12) and eNAMPT serves as a therapeutic target in both breast cancer (13) and prostate cancer (14, 15). A humanized eNAMPT-neutralizing monoclonal antibody (mAb), ALT-100 effective in blocking eNAMPT/TLR4 signaling (16) and reducing severity of preclinical models of organ inflammation and fibrosis (Sammani et al. 2023; Casanova et 2025), reduced the severity of RILF by suppressing the release of inflammatory cytokines. Our former studies in a murine model of metastatic breast cancer in a hind-limb demonstrated that sustained morphine administration induces osteolysis and hypersensitivity within the ipsilateral femur through the activation of TLR4 (8). Bone loss through the process of osteolysis involves the activation of the RANK receptor on osteoclasts by RANKL, a protein secreted by tumor cells and the osteoblastic cell line (8). Nuclear Factor kappa *β* (NFk-*β*) is the downstream mediator of RANK receptor which drives the formation and activity of osteoclasts, the cells responsible for bone resorption. There is a known link between RANK-RANKL pathways and TLR4 which is highly expressed in immune cells within the bone-tumor microenvironment to ultimately increase NFk-*β*-driven osteoclast production and osteolysis, leading to cancer-induced bone pain (8, 13). However, the contribution of eNAMPT to the RANK-TLR4 pathways in the bone environment, including during metastatic bone disease has not been investigated.

The current study determined whether ALT-100, the eNAMPT-neutralizing mAb, could attenuate metastatic breast cancer-induced bone pain as well as alter bone loss using a murine model of cancer-induced bone pain. Specifically, we aimed to understand the expression of eNAMPT in E0771 breast adenocarcinoma cells and ALT-100's role in its inhibition as well as determining the relationship between eNAMPT and osteolysis. Finally, in a murine metastatic cancer model, we tested the efficacy of eNAMPT inhibition via ALT-100 in decreasing cancer induced bone pain (CIBP) and mitigating bone loss.

## 2 Materials and methods

### 2.1 Drugs

ALT-100 was obtained from Aqualung Therapeutics Corporation (Tucson, AZ) and was intraperitoneally (ip) administered at a concentration of 4 mg/kg solubilized in Saline on Day 7, a week after CIBP surgery. Saline was used as vehicle control (10 mL/kg, ip). In the *in vitro* experiments, ALT-100 was administered at either 10ug/mL or 20ug/mL concentration; media was used as control. For the osteoclastogenesis assay, eNAMPT was acquired (Peprotech, Cranbury, New Jersey) and administered at a concentration of 2ug/mL, dissolved in media.

### 2.2 Animals

All studies used female C57BL/6J mice (Jackson Laboratories) aged 7 to 9 weeks at the time of surgery (*n* = 7–14 animals per treatment group) (8). This was vital to confirm immunocompatibility with the E077 L mammary adenocarcinoma cell line (i.e., syngeneic). The choice to use female animals was considered based on the prevalence of breast cancer in females. Animals were maintained on a 12-hour light or dark cycle in a climate-controlled room and were provided access to food and water *ad libitum*. Animals were weighed on days 0 (before surgery), 7, 10, and 14. The animals were monitored for any sort of clinical signs of morbidity, including but not limited to displaced patella, paralysis, rapid weight loss (>20% in 1 week), and full cortical fracture. If such incidence occurred, the animal would be removed from the study. All procedures were performed during the 12-h light cycle and according to the policies and recommendations of the International Association for the Study of Pain, the NIH guidelines for laboratory animals, with IACUC approval from the University of Arizona (Protocols: 19–600). Animal numbers required to achieve statistical power for each assay were determined by G.Power 3.1 in alignment with NIH policy (NOT-OD-15-102) such that differences of 20% were detected with 80% power at a significance level of 0.05. The total number of animals used was 54. Animals were randomly assigned groups, and all behavioral testing was performed using a blinded-to-treatment protocol.

### 2.3 Cancer-induced bone pain surgery

Intramedullary injections of EO771 cells were performed as previously described (1, 17–19) Briefly, mice were anesthetized with 5% isoflurane in 100% O2 (1.8–2L flow rate). A lateral incision was made on the right hind leg over the femur to expose the thigh muscle. An arthrotomy was conducted, and the condyles of the distal femur were revealed. A hole was drilled in the intercondylar space to access the medullary cavity. EO771 breast adenocarcinoma cells (8  ×  10^4^) in 5 µL volume of OPTI-MEM were injected into the cavity using an injection cannula fixed by plastic tubing to a 20 µL Hamilton Syringe. SHAM animals only received OPTI-MEM. The Faxitron x-ray confirmed the placement of the cannula before the cells were injected. After injection, the hole in the distal femur was sealed using bone cement. The muscle was closed using 5–0 Vicyl suture, and the skin incision was sealed using wound clips. Animals were given 7 days of recovery before behavioral testing was conducted. Wound clips were also removed 7 days after surgery. Based on the Faxitron x-rays, animals that had an early sign of radiographic fracture were removed from the study and excluded from data analysis (8).

### 2.4 Spontaneous pain behaviors

Individual mice were placed into a PlexiGlas chamber with a mesh floor. They were then allowed to acclimate for 30 min before testing. Over 2 min, animals were observed for both guarding and flinching behaviors as previously described (12, 20). To summarize, flinching is recognized by the rapid flexing of the ipsilateral hind paw, while the animal is at rest. In addition, if the mouse shook its foot while walking, this was also considered a flinch. Guarding is characterized by the amount of time the animal holds its ipsilateral hind paw into a retracted position near or under the torso, without flinching. Throughout the 2-minute period, animals often will display a combination of both flinching and guarding. Flinching represents spontaneous pain while guarding indicates ongoing pain. All mice were baselined for these behaviors on day 0 prior to surgery. Post-operative pain baselines were measured on day 7, prior to injection with ALT-100 or Saline, then re-measured 2 h after the administration of ALT-100 or Saline. On days 10 and 14 post-surgery animals were reexamined for pain behaviors. Behaviors were analyzed by treatment-blinded trained examiners. The same behaviorists were used for the entirety of the study to ensure consistency and reliability.

### 2.5 Radiographic analysis

Mice were placed under anesthesia using 5% isoflurane in 100% O2 (flow rate 1.8–2L/min) while being imaged using the digital Faxitron (UltraFocus Faxitron Bioptics Tucson, AZ) (8). Images for each mouse were taken on days 0 (before surgery), during cannula placement during surgery, then days 7, 10, and 14 post-surgery. The femur was then evaluated using a 5-point bone rating scale by 4 independent and blinded observers (1, 21). The bone scoring scale is rated as so: 0 = normal bone: 1 = 1 to 3 lesions with no fracture; 2 = 4 + lesions with no fracture; 3 = unicortical, full-thickness fracture; and 4 = bicortical, full-thickness fracture. Any animals with full cortical fractures before day 14 were euthanized, and their data were excluded from statistical analysis.

### 2.6 Collection of serum samples

On Day 14, 7 days after either ALT-100 or Saline injection, animals were anesthetized under 5% isoflurane, then whole blood samples were collected from the heart's left ventricle and transferred into microcentrifuge tubes. Samples were centrifuged at 3,500 g for 15 min at 4 °C to separate serum. Serum samples were transferred into new microcentrifuge tubes, then flash-frozen in liquid nitrogen and stored at −80 °C until usage.

### 2.7 eNAMPT ELISA assay

Plasma and cell supernatant levels of eNAMPT, IL-6, IL-1b, and TNF-a were measured 50 uL in an electrochemi-luminescent multiplex immunoassay panel on a meso-scale ELISA platform (Meso Scale Diagnostics, U-PLEX Personalized Multiplexing, Rockville, Maryland, USA) as we previously reported (22–24) Briefly, each biotinylated antibody (10 ug/mL, specific for each analyte, was mixed with a different linker for each analyte and incubated for 30 min at 25 °C. The reaction was terminated with 200uL of free biotin solution and 600 uL of the 10x U-PLEX linked biotinylated antibody solution with 50 uL of coating solution was added to each well in 96 well plate and incubated for 1 h (800 rpm shaking, 25 °C). After washing (TBS-T), each well was supplemented with 50 uL/well of 1x detection antibody solution, incubated for 1 h, washed and supplemented with 2x Read Buffer T followed by plate imaging and calculation of the absolute concentration values based on standards. eNAMPT ELISA (Cat. No. AG-45A-0006YTP-KI01) was performed using assay obtained from AdipoGen according to the manufacturer-specific protocol.

### 2.8 Cell culture

E0771 breast adenocarcinoma cells were gifted by Kathryn Visser at the Olivia Newton-John Cancer Research Institute Metastasis Research Institute (Victoria, Australia). RAW 264.7 murine macrophage cells were obtained from the American Type Culture Collection (Manassas, VA). EO771 cells were maintained in Dulbecco modified Eagle medium (Mediatech Manassas, VA) supplemented with 10% fetal bovine serum, 25 mM HEPES buffer, 100 IU penicillin, and 100 µg/mL streptomycin. RAW 264.7 cells were maintained in Dulbecco-modified Eagle medium supplemented with 10% fetal bovine serum. Cells were maintained in a 5% CO_2_ incubator at 37 °C. Cells were passaged every 4 days or as needed, and cells used in all experiments were in the 10 to 20 passage range.

### 2.9 eNAMPT ELISA assay in E0771 cancer cell supernatants

E0771 cells were plated at a density of 3.0 × 10^5^ per well in a 6-well plate. After they reached 70%–80% confluency, the cells were grown in the presence or absence of ALT-100 (10 μg/mL) or media for 12, 24 and 48 h. The media was collected and subjected to eNAMPT ELISA. In specific experiments, E0771 cells were challenged by eNAMPT secretion agonists: LPS (TLR4 agonist), diABZi (STING agonist). The cells were lysed in in ice-cold lysis buffer (20 mM Tris-HCl, 50 mM NaCl, 2 mM MgCl_2_ × 6H_2_O, 1% v/v NP40, 0.5% v/v sodium deoxycholate, 0.1% v/v SDS; pH 7.4) supplemented with protease and phosphatase inhibitor cocktail (Halt™ Protease and Phosphatase Inhibitor Cocktail, ThermoScientific), then centrifuged at 12,000xg for 10 min at 4 °C. The supernatant was subjected to eNAMPT ELISA by the MSD platform.

### 2.10 Osteoclastogenesis assay on RAW 264.7 cells

RAW 264.7 macrophage cells were plated at a density of 10^4^ cells per well in a 96-well plate to study osteoclast-like differentiation (25). Cells were then immediately treated with different drug combinations: media, eNAMPT (2 ug/mL), ALT-100 (10 ug/mL), 1:1 eNAMPT (2 ug/mL) + ALT-100 (10 ug/mL), and 1:2 eNAMPT (2ug/mL) + ALT-100 (20 ug/mL), with media and drug changes on the third day. The cells were kept in the incubator for 5 days to allow for the osteoclast differentiation process to occur. Tartrate-resistant acid phosphatase (TRAP) levels, a marker for osteoclast maturation, were measured on stained fixed cells on the fifth day using the K-assay TRAP staining kit according to the manufacturer's specifications (Cat. No. KT-008, Kamiya Biomedical Company, Seattle, WA). TRAP activity was determined by measuring the absorbance of the 96-well plates at 540 nm. Images of the RAW 264.7 cells plated in the 96-well plate were captured using an Echo Revolve Microscope on 40x power.

### 2.11 Statistical analysis

Data were analyzed using GraphPad Prism 10.0 software. Data was expressed as mean ± standard deviation, or standard error of the mean as indicated in the individual graphs. Groups were assessed for normality and sphericity before statistical comparison. Statistical significance was assessed via Student`s t-test or one-way ANOVA with Tukey *post hoc* correction, or two-way ANOVA with Bonferroni *post hoc* correction, as indicated in the figure legends. A *p*-value ≤0.05 was considered statistically significant. To limit biased interpretation of data, researchers conducting and analyzing the experiments were blinded to the protocol with another researcher responsible for randomizing and treating subjects to each experiment (e.g., double-blind).

## 3 Results

### 3.1 eNAMPT secretion/release in E0771 cells and E0771-challenged mice is attenuated by ALT-100 mab

In the first experiment, we tested whether EO771 adenocarcinoma cells, used in the murine model of CIBP, spontaneously secreted eNAMPT *in vitro* and whether this secretion was influenced by ALT-100 treatment. EO771 cells were grown under basal conditions in the presence or absence of ALT-100 (10 µg/mL) or media control for 12, 24, and 48 h, after which eNAMPT concentrations in the culture supernatants were quantified. EO771 cells secreted eNAMPT in a time-dependent manner, with maximal extracellular levels observed at 48 h **(**Figure 1A). Treatment with ALT-100 produced an approximately 35% reduction in measurable extracellular eNAMPT at 48 h. Although suggestive of eNAMPT autocrine signaling, this reduction did not reach statistical significance(Figure 1A). Stimulation of EO771 cells with either the STING agonist DIABZi or LPS showed time-dependent increases in eNAMPT secretion that were largely unaffected by ALT-100 (Figure 1B). Under basal conditions, other inflammatory markers, including IL-1β, TNF-α, and IL-6, were not spontaneously released by EO771 cells (Figure 1C). When stimulated with either the STING agonist DIABZi or LPS, only IL-1β showed time-dependent increases and was unaffected by ALT-100 (Figure 1C). Day 14 serum from female C57BL/6J mice challenged with femur injection of E0771 murine breast cancer cells show increased eNAMPT levels which were attenuated in mice receiving 4 mg/kg ALT-100 mAb (Figure 1D).

**Figure 1:**
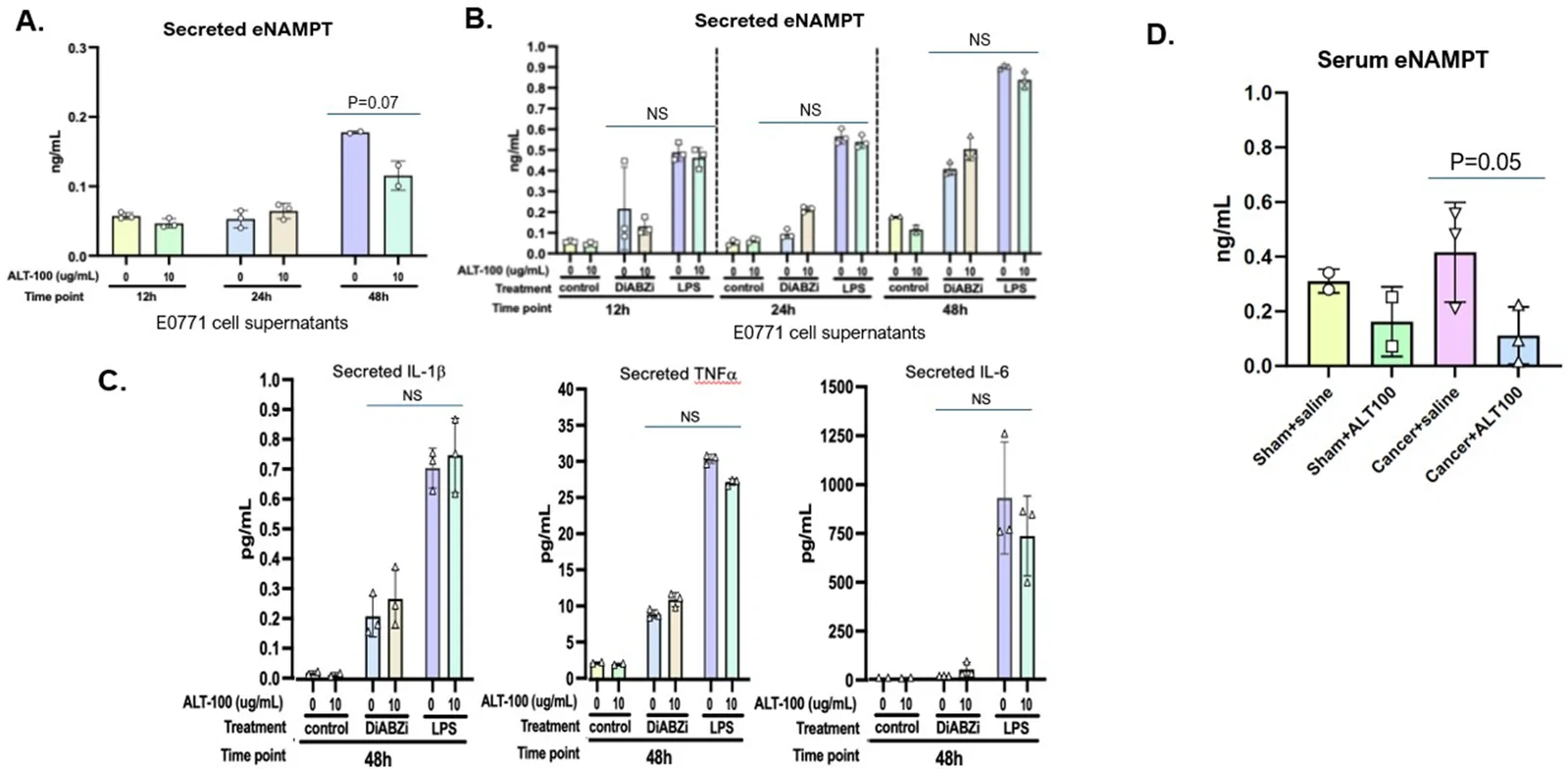
*In vitro* and *in vivo* eNAMPT secretion from E0771 cells and E0771-challenged mice: attenuation by the ALT-100 mab. **(A)** E0771 cells were cultured under basal conditions in the presence of ALT-100 (10 µg/mL) or media control for 12, 24, or 48 h and culture supernatants analyzed using the MSD ELISA platform for eNAMPT and inflammatory cytokines. EO771 cells constitutively secreted eNAMPT in a time-dependent manner, with maximal secretion observed at 48 h. ALT-100 reduced measurable extracellular eNAMPT (mean ng/mL ± SEM, *n* = 3/group) by approximately 35% at 48 h, although this difference did not reach statistical significance (*p* = 0.07, unpaired Welch t-test). **(B)** E0771 cell cultures were stimulated with either the STING agonist diABZi or lipopolysaccharide (LPS) which produced time-dependent increases in eNAMPT secretion that were largely unaffected by incubation with ALT-100 (mean ng/mL ± SEM, *n* = 3/group) (ns = not significant). **(C)** Under basal conditions, EO771 cells did not spontaneously secrete detectable levels of IL-1β, TNF-α, or IL-6. Shown are findings at 48 h with diABZi inducing IL-1β and TNF-αrelease and LPS inducing IL-1β, TNF-α, and IL-6 release. Findings which were not altered by ALT-100 incubation (mean ± SEM in pg/mL, *n* = 3/ group, Two-way ANOVA) (ns = not significant). **(D)** Day 14 serum from female C57BL/6J mice challenged with femur injection of E0771 murine breast cancer cells was analyzed by the MSD ELISA platform. Compared to the Sham + Saline and the Sham + ALT-100 groups without E0771 murine breast cancer cell exposure, the Cancer + Saline group exhibited increases in eNAMPT which were attenuated in mice receiving 4 mg/kg ALT-100 mAb (*p* = 0.05).

### 3.2 ALT-100 inhibits eNAMPT-activated TLR-4 osteoclastogenesis *in vitro*

Next, we assessed the ability of ALT-100 to inhibit osteoclastogenesis *in vitro* using RAW 264.7, an osteoclast precursor cell line. Cultured RAW 264.7 murine macrophages were separated into five groups exposed to different drug combinations: media, eNAMPT (2 ug/mL), ALT-100 (10 ug/mL), 1:1 eNAMPT (2 ug/mL) + ALT-100 (10 ug/mL), and 1:2 eNAMPT (2ug/mL) + ALT-100 (20 ug/mL), with media and drug changes on the third day. Levels of tartrate-resistant acid phosphatase (TRAP), a marker for osteoclast maturation (10, 17), were measured in stained fixed cells on the fifth day via absorbance at 540 nm (Figure 2A). Exposing the RAW 264.7 cells to eNAMPT treatment significantly increased TRAP staining compared to media alone (Figure 2C), indicating eNAMPT's ability to induce osteoclastogenesis. Exposing the RAW cells to ALT-100 alone showed no changes in TRAP activity compared to the media (Figure 2C). When exposing the RAW cells to ALT-100 + eNAMPT, it was found that TRAP activity was significantly less than the eNAMPT alone group (Figure 2C). This indicates that ALT-100 inhibits eNAMPT-enhanced osteoclastogenesis in both combined therapy groups (Figure 2C).

**Figure 2:**
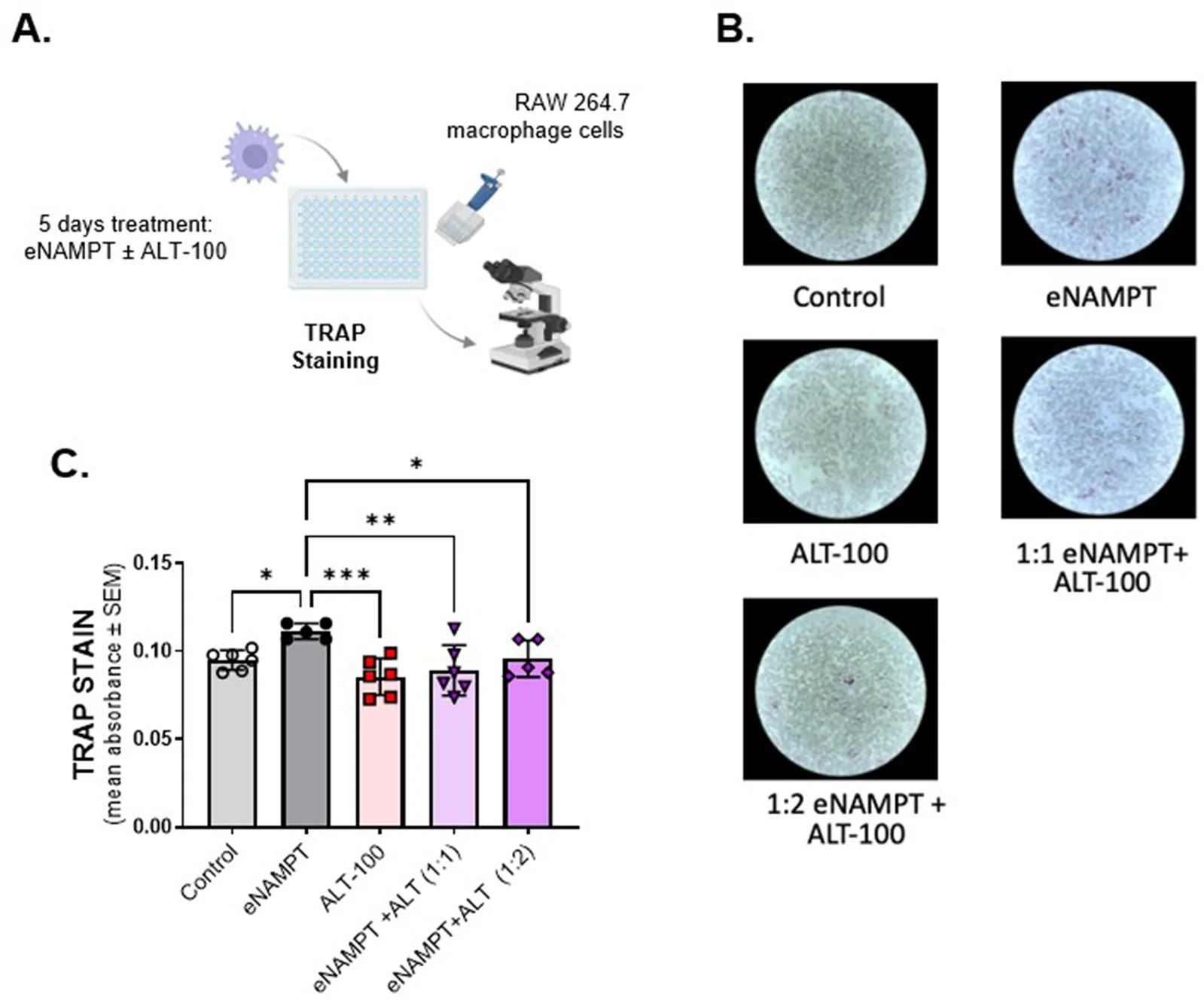
ALT-100 neutralizes eNAMPT-enhanced osteoclastogenesis in RAW264.7 cells. RAW264.7 cells were treated with recombinant eNAMPT (2 µg/mL, Peprrotech) in the presence or absence of ALT-100 (10 µg/mL or 20 µg/mL) for 5 days to induce differentiation, followed by staining for tartrate-resistant acid phosphatase (TRAP). **(A)** The panel shows the experimental setting. **(B)** Representative TRAP-stained images of RAW264.7 cells captured on Echo Revolve microscope at 40x power. **(C)** Quantification of TRAP activity in conditioned media is shown. Exposing RAW264.7 cells to eNAMPT significantly increased TRAP activity compared to media control (media vs. eNAMPT: *p* = 0.0113). Treatment with ALT-100 alone did not significantly change TRAP activity compared to media (media vs. ALT-100: *p* = 0.1077). Co-treatment with ALT-100 and eNAMPT significantly reduced TRAP activity compared to eNAMPT alone at both doses (eNAMPT vs. 1:1 eNAMPT + ALT-100: *p* = 0.0011; eNAMPT vs. 1:2 eNAMPT + ALT-100: *p* = 0.0196). In contrast, comparisons between ALT-100 and combined treatment groups showed no significant differences (ALT-100 vs. 1:1 eNAMPT + ALT-100: *p* = 0.5246; ALT-100 vs. 1:2 eNAMPT + ALT-100: *p* = 0.0969). There was also no significant difference between the two combined treatment groups (1:1 eNAMPT + ALT-100 vs. 1:2 eNAMPT + ALT-100: *p* = 0.2766). Data are expressed as mea*n* ± SEM and analyzed by one-way ANOVA [F(4,23) = 5.41, *n* = 5–6 cultures/condition] with Fisher's LSD *post hoc*. *p < 0.05, **p < 0.01, ***p < 0.001,****p < 0.0001, ns = not significant.

### 3.3 ALT-100 treatment significantly reduced cancer-induced osteolysis

To evaluate the effects of ALT-100 on tumor-related bone loss, radiographs obtained at baseline (Day 0, prior to surgery) and on Days 7, 10, and 14 after inoculation were scored and compared (Figure 3A). The baseline radiographs were then compared with radiographs obtained on Day 14 to determine the extent of osteolysis using the 5-point scale described in Method section (1, 12, 21). (Figure 3B). Animals inoculated with cancer cells exhibited significantly higher bone scores compared with SHAM inoculated control groups, regardless of treatment (Figure 3C), indicative of cancer-induced bone loss. However, the Cancer group treated with ALT-100 exhibited significantly lower scores compared to Saline-treated Cancer animals (Figure 3C). These results indicate that ALT-100 significantly reduces tumor-induced osteolysis.

**Figure 3:**
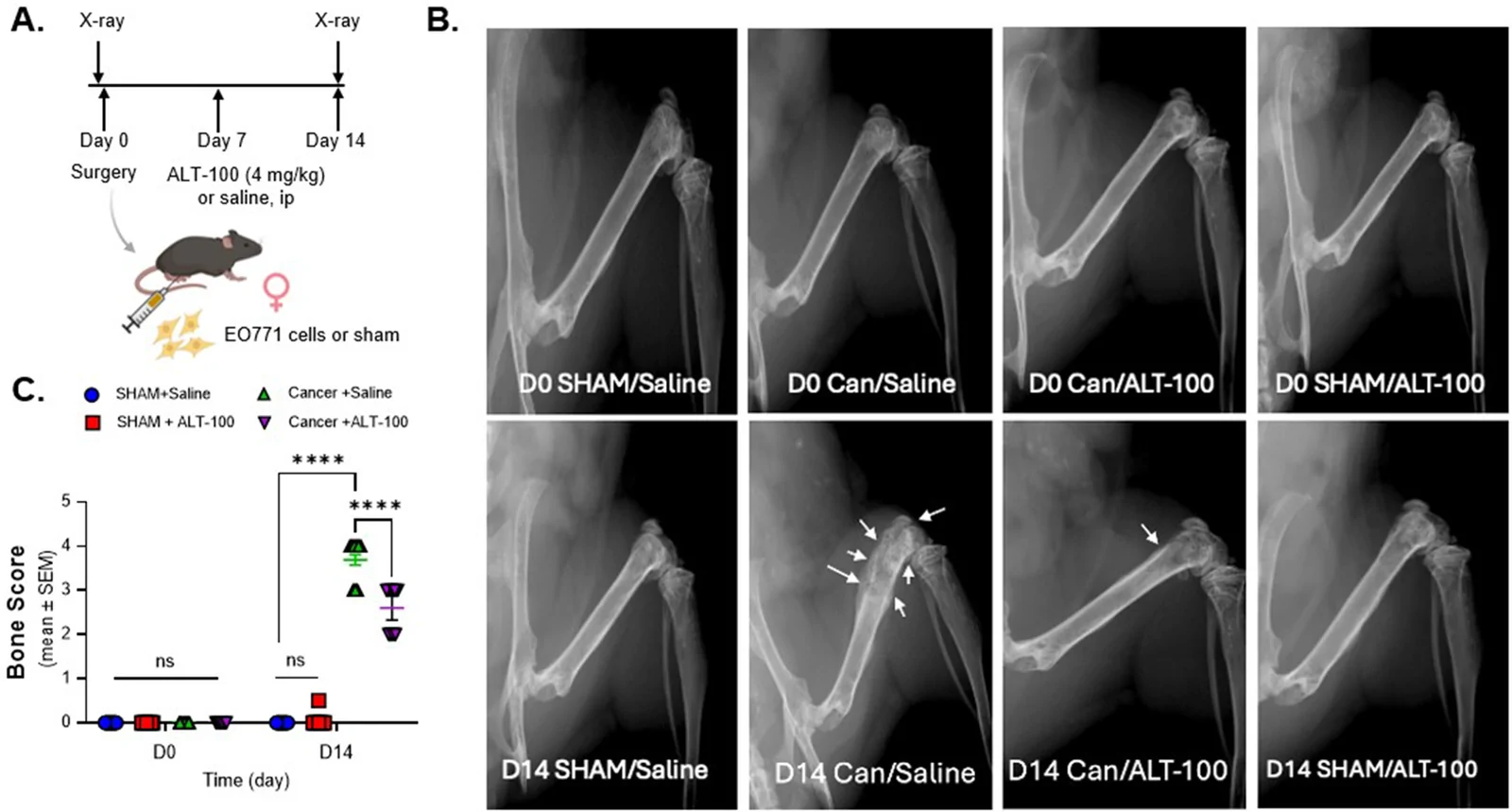
ALT-100 significantly decreased osteolysis in mouse models of CIBP. CIBP surgery, along with SHAM control was performed on female C57BL/6J mice using EO771 cells. After 7 days of recovery, mice were ip injected with ALT-100 (4 mg/kg) or Saline (10 mL/kg, ip) on Day 7. Radiographs were obtained before the surgery on Day 0 and post-surgery on Day14 to evaluate cancer-induced bone lesions. **(A)** The schema of the experimental setting. **(B)** Representative radiographs of the right femur before surgery (Day 0) and on Day 14 post-surgery. White arrows indicate the areas of osteolysis and bone degeneration. **(C)** Quantification of bone scores (*n* = 9–16 per treatment group). Bone scores were determined on a 5-point scale by blinded observers, where 0 = normal bone, 1 = 1–3 lesions with no fracture, 2 = ≥4 lesions with no fracture, 3 = unicortical, full-thickness fracture, and 4 = bicortical, full-thickness fracture. At Day 0, no significant differences were observed among groups (all *p* > 0.9999). At Day 14, Cancer + Saline mice exhibited severe osteolysis compared with SHAM + Saline (mean diff. = −1.584, 95% CI [−1.689 to −1.480], *p* < 0.0001) and SHAM + ALT-100 (mean diff. = −1.584, 95% CI [−1.689 to −1.480], *p* < 0.0001). Importantly, ALT-100 treatment significantly reduced osteolysis in tumor-bearing mice compared with Cancer + Saline (mean diff. = 1.584, 95% CI [1.480 to 1.689], *p* < 0.0001). Data are expressed as mea*n* ± SEM and analyzed by two-way ANOVA [Interaction: F(3,100) = 319.7, *p* < 0.0001; Row Factor: F(1,100) = 903.4, *p* < 0.0001; Column Factor: F(3,100) = 319.7, *p* < 0.0001], followed by Tukey's multiple comparisons test. ****p < 0.0001, ns = not significant.

### 3.4 ALT-100 significantly reduced pain-related behavioral in the murine CIBP Model

To study the effects of ALT-100 on bone-derived pain induced by metastatic breast cancer, we used the established CIBP model (1, 17–19) Flinching and guarding behaviors were measured to determine whether ALT-100 treatment influenced pain-related behaviors (Figure 4A). Flinching and guarding behaviors were recorded for 2 min prior to surgery (Day 0) to confirm all animals were healthy at baseline and failed to detect significant differences between any groups (Figures 4B,C), confirming comparable baseline pain behavior. After confirmation, EO771 breast adenocarcinoma cells or media control (SHAM) were next injected into the intramedullary space of the femur of female C57BL/6J mice and allowed to seed for 7 days. On Day 7 post-cancer inoculation, flinching and guarding behaviors were again recorded for 2 min to establish a post-operative baseline and to compare SHAM versus cancer animals prior to treatment. Cancer + ALT-100 exhibited significantly greater flinching compared with SHAM + Saline (Figure 4B) and SHAM + ALT-100 (Figure 4B), while Cancer + Saline did not differ significantly from either SHAM group (Figure 4B). Guarding behaviors showed no significant differences between any groups at this pre-injection time point (Figure 4C).

**Figure 4:**
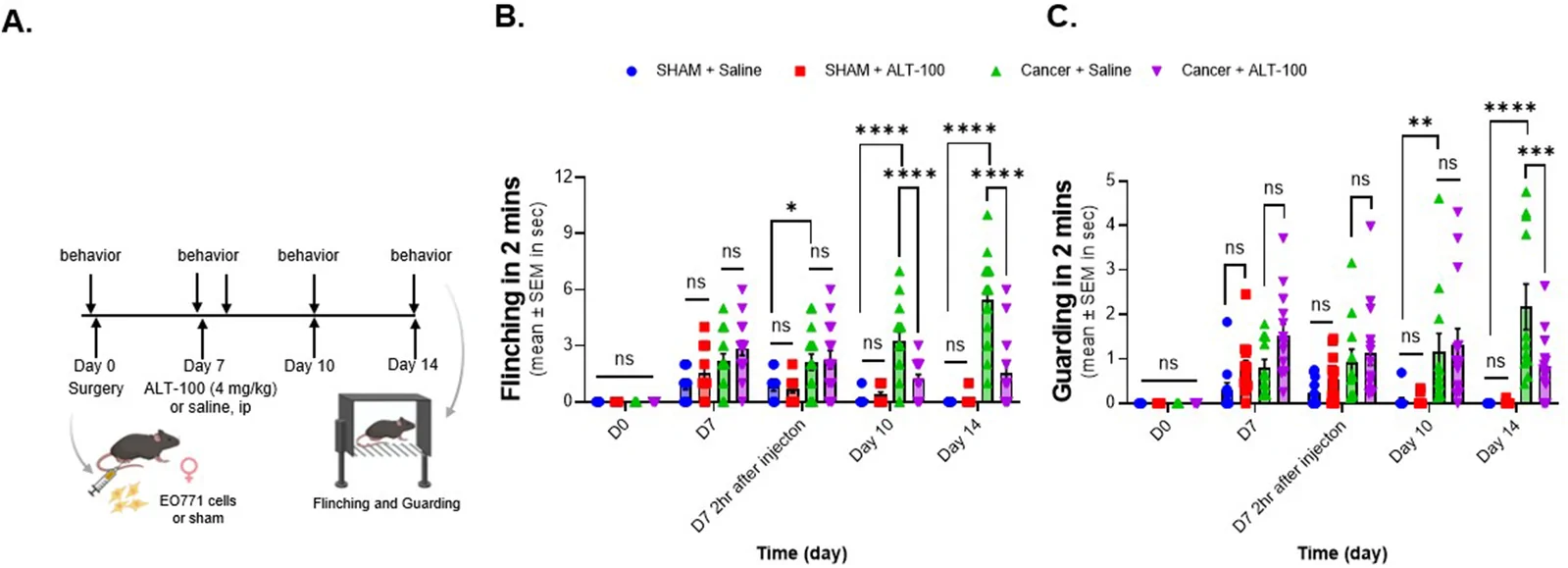
Administration of ALT-100 produces an analgesic effect in a murine model of cancer-induced bone pain. CIBP surgery, along with SHAM control was performed on female C57BL/6J mice using EO771 cells. ALT-100 (4 mg/kg, i.p.) or Saline (10 mL/kg, i.p.) were injected on Day 7. Spontaneous pain behaviors (flinching and guarding) were assessed on Day 0 before surgery, on Day 7 before and 2 h after injections, on Day 10 and 14. **(A)** The experimental setting. **(B)** At D0, all groups showed similar flinching behavior before treatment initiation (all comparisons: *p* > 0.9999). At D7, Cancer + ALT-100 mice exhibited significantly elevated flinching compared with SHAM + Saline (*p* = 0.0008) and SHAM + ALT-100 (*p* = 0.0319). Two hours post-injection on D7, ALT-100 treatment did not significantly reduce flinching compared with Cancer + Saline (*p* = 0.9897), though cancer groups remained elevated compared with SHAMs (Cancer + Saline vs. SHAM + Saline: *p* = 0.0457; Cancer + ALT-100 vs. SHAM + Saline: *p* = 0.0232). At D10, Cancer + ALT-100 demonstrated significantly reduced flinching compared with Cancer + Saline (*p* < 0.0001), while differences between cancer and SHAM groups persisted (Cancer + Saline vs. SHAM + Saline: *p* < 0.0001; Cancer + Saline vs. SHAM + ALT-100: *p* < 0.0001). At D14, Cancer + ALT-100 exhibited significantly lower flinching compared with Cancer + Saline (*p* < 0.0001), demonstrating the sustained analgesic effect of ALT-100. Data are shown as mean of the time spent flinching in seconds ± SEM (*n* = 7-13 per group) and was analyzed by two-way ANOVA with Bonferroni *post hoc* (Interaction: F(12,255) = 10.45, *p* < 0.0001). **(C)** At D0, no significant differences in guarding behaviors were observed between groups (all comparisons: *p* > 0.9999). At D7, Cancer + ALT-100 mice showed significantly elevated guarding compared with SHAM + Saline (*p* = 0.0005). Two hours post-injection on D7, ALT-100 treatment partially reduced guarding, though levels remained elevated compared with SHAMs (Cancer + ALT-100 vs. SHAM + Saline: *p* = 0.0197). At D10, Cancer + ALT-100 mice exhibited reduced guarding relative to Cancer + Saline (*p* = 0.0025) and SHAM controls (Cancer + ALT-100 vs. SHAM + Saline: *p* = 0.0003; Cancer + ALT-100 vs. SHAM + ALT-100: *p* = 0.0001). At D14, Cancer + ALT-100 significantly decreased guarding compared with Cancer + Saline (*p* < 0.0001), while SHAM groups remained unchanged (SHAM + Saline vs. SHAM + ALT-100: *p* > 0.9999). Data are expressed as mean of the time spent in guarding in seconds ± SEM (*n* = 7–13/treatment) and analyzed by two-way ANOVA with Bonferroni *post hoc* (Interaction: F(12,225) = 4.432, *p* < 0.0001). *p < 0.05, **p < 0.01, ***p < 0.001, ***p < 0.0001, ns = not significant.

Two hours after injection on Day 7, we compared SHAM + Saline vs. SHAM + ALT-100 and Cancer + Saline vs. Cancer + ALT-100 to determine whether ALT-100 altered pain within each condition. Both Cancer + Saline (Figure 4B) and Cancer + ALT-100 (Figure 4B) showed significant increases in flinching compared with SHAM + Saline. Cancer + ALT-100 also differed from SHAM + ALT-100 (Figure 4B) demonstrating that animals with cancer exhibited pain compared to sham treatment. However, there were no differences between Cancer + ALT-100 and Cancer + Saline at this early time point (Figure 4B).

By Day 10, Cancer + Saline showed significantly more flinching than both SHAM groups (Figure 4B**)**. Importantly, the Cancer + ALT-100 group demonstrated significantly reduced flinching compared with Cancer + Saline (Figure 4B). By Day 14, the flinching behavior in Cancer + Saline remained significantly elevated relative to SHAMs groups (Figure 4B), while Cancer + ALT-100 exhibited markedly reduced flinching compared with Cancer + Saline (Figure 4B), although remaining slightly higher than SHAM groups (Figure 4A). SHAM + Saline and SHAM + ALT-100 did not differ significantly at any time point **(**Figure 4B).

For guarding behaviors (Figure 4C), Day 0 baselines showed no significant differences between groups. At Day 7 pre-injection, there were no significant differences observed between groups. Two hours post-injection on Day 7, Cancer + ALT-100 showed significantly more guarding compared with SHAM + Saline, while Cancer + Saline did not differ from SHAM groups, and Cancer + ALT-100 did not differ from Cancer + Saline. By Day 10, Cancer + Saline exhibited significantly elevated guarding compared with SHAM + Saline, SHAM + ALT-100, as well as Cancer + ALT-100. On Day 14, Cancer + Saline continued to show more guarding compared with SHAM + Saline or SHAM + ALT-100, while Cancer + ALT-100 was reduced relative to Cancer + Saline. SHAM + Saline and SHAM + ALT-100 remained unchanged across all time points. Overall, cancer-inoculated mice administered ALT-100 demonstrated significantly reduced flinching and guarding behaviors through Day 10 and Day 14 compared to mice administered Saline.

## 4 Discussion

Cancer induced bone-pain (CIBP) is a debilitating condition associated with metastatic cancer, and is characterized by inflammatory and neuropathic mechanisms that contribute to chronic pain and bone degradation (1, 2). A multitude of factors contribute to the pain including tumor invasion, inflammatory cytokine release, and osteoclast activation resulting in bone loss, all leading to a decreased quality of life in metastatic cancer patients. Currently, opioid analgesics remain the standard treatment for CIBP. However, opioid use enhances bone loss in both mice and humans (26–30), and potentially enhances tumor growth (31–33) The well-known escalating opioid doses required due to analgesic tolerance triggers and increases inflammation over time with long-term adverse effects such as respiratory depression and addiction (8, 34). Given the negative effects of opioid use, there is an urgent need for non-opioid-based therapeutics that effectively alleviate CIBP with minimal adverse effects.

In this study, we demonstrated that ALT-100, a humanized mAb targeting eNAMPT, attenuates osteolysis, osteoclastogenesis, and pain behaviors in a murine model of metastatic breast cancer. Our findings are summarized in Figure 5. We first confirmed that E0771 breast cancer cells synthesize and secrete eNAMPT, corroborating prior research that reported eNAMPT overexpression in various malignancies (13–15, 35). ELISA results of E0771 cell lysate and supernatant revealed significant time-dependent secretion/release of eNAMPT levels maximal at 48 h which was attenuated by treatment with ALT-100 consistent with the eNAMPT autocrine signaling. Furthermore, Day 14 serum from E0771-femur challenged mice showed increased eNAMPT levels which were attenuated in mice receiving 4 mg/kg ALT-100 mAb consistent with a systemic innate immune response to cancer invasion in bone. Using RAW 264.7 undifferentiated macrophages, we demonstrated that ALT-100 significantly inhibited eNAMPT-driven TRAP positive osteoclast formation, indicating blockade of osteoclastogenic signaling. Radiographic analysis showed significantly lower bone scores in Cancer + ALT-100 mice than in Cancer + Saline controls, demonstrating reduced osteolysis. Finally, through behavioral analysis, we demonstrated by Day 14, ALT-100 significantly reduced flinching and guarding behaviors compared to Cancer + Saline animals, confirming antinociceptive effects across two pain behaviors.

**Figure 5:**
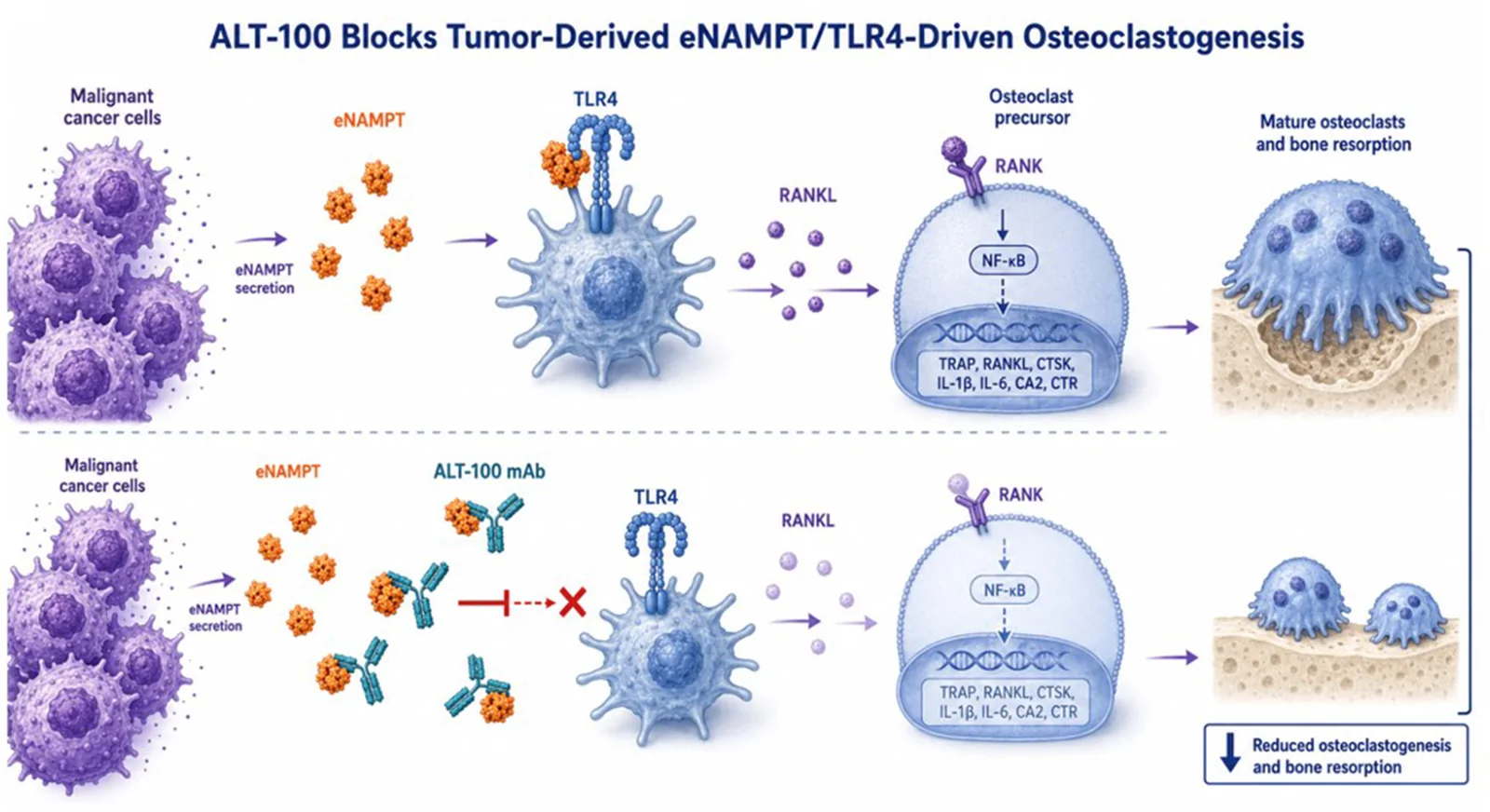
Proposed mechanism of eNAMPT-induced hypersensitivity and enhanced osteolysis via ALT-100 mab inhibition. As the ALT-100 mAb prevents eNAMPT binding and activation of the TLR4 receptor and downstream NF-*κ*B signaling (16), we speculate that in the bone tumor microenvironment, where inflammation is elevated, the administration of ALT-100 mAb inhibits eNAMPT/TLR4 signaling to reduce the production of DAMPs and inflammatory cytokines such as IL-6, TNF*α*, and IL-1β (8, 36, 37) which exert differential effects on osteoclasts and osteoblasts. Chronic inflammation negatively impacts osteoblastic differentiation and activity, contributing to impaired bone formation and bone degradation. Acting through multiple receptors, including IL-6R and TNFR, the upregulation of genes contributes to enhanced osteoclast activity, whereas downregulation of osteoblastic genes leads to reduced osteoblast differentiation (8). These effects are predicted to promote inflammatory pain states and increase the risk of pathological fracture. Administration of ALT-100 is theorized to block these eNAMPT-mediated effects, reducing osteolysis and hypersensitivity consistent with our radiographic and behavioral findings showing decreased osteolysis and reduced flinching and guarding in ALT-100 treated mice. IL-1β, interleukin 1*β*; IL-6, interleukin 6; TNF*α*, tumor necrosis factor alpha; TLR4, toll-like receptor-4; NF-*κ*B, nuclear factor kappa B; ALT-100 monoclonal antibody; IL-6R, interleukin-6 receptor; TNFR, tumor necrosis factor receptor. Image created with assistance from the image generation feature in OpenAI's ChatGPT.

Our findings are the first to demonstrate that direct targeting of eNAMPT with the ALT-100 mAb serves to interrupt a key upstream mechanism of CIBP. Previous studies have implicated TLR4–NF-*κ*B signaling in osteoclastogenesis and bone resorption, with proinflammatory cytokines such as TNF-α and IL-1β acting downstream. Extracellular NAMPT has been described as a DAMP capable of binding TLR4 and activating downstream NF-*κ*B signaling, thereby amplifying inflammatory responses (10, 16, 35). By neutralizing eNAMPT, ALT-100 prevents/attenuates this TLR4 activation and downstream signaling. As illustrated in Figure 5, we propose that within the inflammatory bone tumor microenvironment, eNAMPT/TLR4 signaling may contribute to increased production of inflammatory mediators, including IL-6, TNF-α, and IL-1β, which can promote osteoclast activity while negatively affecting osteoblast differentiation and bone formation (8, 36, 37). This imbalance in bone remodeling may contribute to tumor-associated osteolysis and, together with inflammatory signaling within the tumor-bone microenvironment, promote nociceptive sensitization and cancer-induced pain. This proposed mechanism of upstream blockade aligns with and extends former results showing that interrupting inflammatory signaling upstream produces both anti-osteolytic and antinociceptive effects (8), Our data also diverge from earlier reports focused on cytokine or receptor antagonism by highlighting eNAMPT as the initiating DAMP and TLR4 ligand (10) and demonstrating that its neutralization mitigates osteoclastogenesis and pain. Importantly, ALT-100 targets an upstream signal that is known to result in a cascade of upregulated pro-inflammatory cytokines suggesting that such a therapy is more likely to reduce pain than selectively targeting an individual cytokine.

Our findings provide experimental support for this proposed mechanism. We demonstrated that E0771 breast cancer cells synthesize and secrete eNAMPT, that recombinant eNAMPT promotes TRAP-positive osteoclast formation in RAW 264.7 cells, and that ALT-100 inhibits this eNAMPT-induced osteoclastogenic response. *In vivo*, ALT-100 treatment was associated with reduced radiographic osteolysis as well as reduced flinching and guarding behaviors. Together, these findings suggest that neutralization of eNAMPT may connect reduced osteoclastogenesis and bone destruction with attenuation of pain-related behaviors, consistent with the proposed mechanism shown in Figure 5.

eNAMPT has been implicated in other inflammatory (24, 36), organ fibrosis (11, 14, 22) and cancer conditions (13, 15), via its function as a DAMP (damage-associated molecular pattern protein) activating TLR4 signaling and amplifying innate immune responses. Our work adds breast cancer-induced bone pain to this growing list and suggests that eNAMPT is a central mediator of tumor-associated bone destruction and pain. ALT-100 studies both validates the role of eNAMPT in CIBP pathobiology and identifies ALT-100 as a novel therapeutic tool in multiple pain disorders and cancer models.

Beyond cancer disorders, numerous studies have reported the connection between eNAMPT and various inflammatory and metabolic diseases such as diabetes (38), atherosclerosis, arthritis, lupus vasculitis (39) and inflammatory bowel disease (40), many of them are associated with pain and pain disorders. Preclinical and clinical data suggest that eNAMPT is involved in the pathogenesis of these conditions, mainly by promoting inflammatory and profibrotic pathways with cellular phenotypic switching and endothelial/epithelial cell transition to apoptosis-resistant mesenchymal cells (22, 41). In osteoarthritis, eNAMPT can promote tissue degradation and increase the expression of a variety of proteases. Pecchi et al. showed that eNAMPT stimulates NGF expression and release by chondrocytes in a model of osteoarthritis, suggesting possible involvement in osteoarthritis-associated pain (40, 42). In clinical studies, increased eNAMPT level was observed in the synovial fluid of patients with osteoarthritis, and it was positively associated with pain scores (43, 44). Similar to osteoarthritis, increased synovial level of eNAMPT was detected in patients with rheumatoid arthritis. There was a positive correlation between eNAMPT level and severity/duration of rheumatoid arthritis with eNAMPT levels particularly high in patients showing radiographic signs of joint damage (45).

Collectively, these results suggest targeting eNAMPT to therapeutically inhibit osteoclastogensis as a promising therapy. The intracellular form of NAMPT possesses enzymatic activity responsible for NAD metabolism and early drug development studies utilizing small molecule inhibitors of NAMPT enzymatic activity to target neoplastic disorders produced positive results in preclinical models but failed in human studies due to low efficacy and unacceptable toxicity (46). The functional effects of extracellular NAMPT (eNAMPT) are independent of its enzymatic activity and intrinsic to its function as a DAMP via TLR4 ligation (10, 35, 40). The humanized eNAMPT-neutralizing ALT-100 mAb exerts beneficial effects in animal models of inflammatory lung injury, radiation-induced lung fibrosis, and prostate cancer cell proliferation (11, 37, 39). Moreover, the safety and tolerability of ALT-100 in healthy volunteers and in Phase 2a clinical trial for the treatment of severe acute lung injury were recently reported (47, 48).

Overall, our results indicate that the ALT-100 mAb offers a targeted, non-opioid strategy to alleviate cancer-induced bone pain by disrupting eNAMPT-driven TLR4 signaling, as well as reducing osteoclastogenesis and osteolysis. As ALT-100 interferes with an extracellular innate immunity signaling mediator rather than intracellular enzymatic activities, this biologic therapy may be especially valuable in patients for whom opioids are ineffective or contraindicated. Further studies should explore optimal dosing, long-term outcomes, and integration with standard cancer therapies. Given the involvement of eNAMPT across malignancies and inflammatory conditions, this approach has potential applicability well beyond breast cancer, offering a promising framework for future non-opioid pain therapeutics.

## Data Availability

The original contributions presented in the study are included in the article/supplementary material, further inquiries can be directed to the corresponding author/s.
